# The interaction between MALAT1 and TUG1 with dietary fatty acid quality indices on visceral adiposity index and body adiposity index

**DOI:** 10.1038/s41598-023-50162-9

**Published:** 2024-01-02

**Authors:** Niloufar Rasaei, Fatemeh Gholami, Mahsa Samadi, Farideh Shiraseb, Alireza Khadem, Mir Saeed Yekaninejad, Solaleh Emamgholipour, Khadijeh Mirzaei

**Affiliations:** 1 Department of Community Nutrition, School of Nutritional Sciences and Dietetics, Tehran University of Medical Sciences (TUMS), P.O. Box 14155-6117, Tehran, Iran; 2 Network of Interdisciplinarity in Neonates and Infants (NINI), Universal Scientific Education and Research Network (USERN), Tehran, Iran; 3 Department of Nutrition, Science and Research Branch, Islamic Azad University, Tehran, Iran; 4 Department of Epidemiology, School of Public Health, Tehran University of Medical Sciences, Tehran, Iran; 5 Department of Clinical Biochemistry, School of Medicine, Tehran University of Medical Sciences, Tehran, Iran; 6 Metabolomics and Genomics Research Center, Endocrinology and Metabolism Molecular-Cellular Sciences Institute, Tehran University of Medical Sciences, Tehran, Iran

**Keywords:** Genetics, Diseases

## Abstract

We aimed to investigate the interaction between the transcript levels of taurine-upregulated gene 1 (TUG1) and metastasis-associated lung adenocarcinoma transcript 1 (MALAT1) and the Cholesterol-Saturated Fat Index (CSI) in relation to the visceral adiposity index (VAI) and body adiposity index (BAI). This cross-sectional study involved 346 women classified as obese and overweight, aged between 18 and 48 years. Dietary intake and the quality of dietary fat were assessed using a validated and reliable 147-item semi-quantitative food frequency questionnaire, with the Cholesterol-Saturated Fat Index (CSI) used as an indicator. Transcription levels of MALAT1 and TUG1 were evaluated through real-time polymerase chain reaction following the criteria outlined in the Minimum Information for Publication of Quantitative standards. Serum profiles were measured using standard protocols. We observed a positive association between transcription level of MALAT1 and VAI in both crude (β = 3.646, 95% CI 1.950–5.341, *p* < 0.001) and adjusted (β = 8.338, 95% CI 6.110–10.566, *p* < 0.001) models. Furthermore, after adjusting for confounders, a significant positive interaction was noted between MALAT1 expression and CSI on BAI (β: 0.130, 95% CI 0.019, 0.240, *p* = 0.022), with a marginal positive interaction observed on VAI (β: 0.718, 95% CI − 0.028, 1.463, *p* = 0.059). It seems that there may be a positive interaction between MALAT1 transcription level and CSI on VAI and BAI among overweight and obese women. However, no associations were seen between TUG1 mRNA level and the above-mentioned outcomes. Further functional studies are still required to elucidate this concept.

## Introduction

One of the main contributors to global non-communicable diseases and mortality is the rising incidence of obesity^1–3^. Estimates suggest that by 2030, one billion people worldwide, comprising one in seven males and one in five females, will be affected by obesity^4^. Obesity affects roughly 21.7% of the adult population of Iran^5^. Additionally, Iranian women (57%) were more likely than men (42.8%) to be overweight or obese^6–8^.

While adipose tissue is widely distributed throughout the body and serves crucial functions in maintaining health, it is essential to recognize it as a highly active endocrine organ^9,10^. Obese individuals are more prone to metabolic disorders due to the increased susceptibility associated with visceral adipose tissue (VAT) compared to subcutaneous adipose tissue (SAT)^11^. The VAT is related to the pathogenesis of insulin resistance, dyslipidemia, and cardiovascular diseases (CVDs)^11,12^. Among obesity indices, visceral adiposity index (VAI) and body adiposity index (BAI) are two novel anthropometric measures that hold the potential to enhance the assessment of obesity, body composition, and the identification of metabolic syndrome^13,14^. Various factors, including low socioeconomic status, a sedentary lifestyle, environmental influences, genetic variations or expressions, molecular mechanisms, and diet, play a role in the etiology of adiposity^15–19^.

To develop effective diagnostic and treatment techniques, a comprehensive understanding of the molecular pathways governing the onset of metabolic syndrome and obesity is imperative^20^. A subclass of non-coding RNAs called long non-coding RNAs (lncRNAs) plays crucial roles in a variety of biological processes because they can regulate gene expression at various levels^21,22^.

Cutting-edge research has revealed that lncRNA dysregulation plays crucial roles in maintaining homeostasis, and control of lipid metabolism, and fat accumulation^20,23,24^. Recent research suggests that the lncRNAs; taurine-upregulated gene 1 (TUG1) and metastasis-associated lung adenocarcinoma transcript 1 (MALAT1) may play regulatory roles in the etiology of obesity-related illnesses and the control of lipid metabolism^25^. There is limited and somewhat conflicting research on the role of MALAT1 in obesity. Recent findings indicate a reduction in MALAT1 expression in white adipose tissue from obese mice. However, the deletion of MALAT1 showed minimal impact on diet-induced growth in adipose tissue and lipid balance in obese animals^25^. On the contrary, distinct research has revealed a significant increase in MALAT1 expression in the medium released by adipose-derived stem cells and omental depots from obese individuals^26^. Another study also showed that mesenteric adipose tissue-derived exosomes from mice on a high-fat diet exhibited elevated MALAT1 expression^27^ Moreover, Ebrahimi et al.^28^ reported that VAT and SAT from obese women had considerably lower mRNA levels of TUG1 in comparison with the controls.

In addition to lncRNAs, the diet has a crucial role in adiposity^29^. All facets of fatty acids in the diet should be taken into account to analyze how the quality of dietary fat consumption relates to obesity and body fat percentage^30^. An innovative dietary self-monitoring tool, proposed by Connor et al., is the Cholesterol-Saturated Fat Index (CSI), which provides information on the cholesterol and saturated fat content of meals^31^. A low CSI index indicates a reduction in saturated fatty acid (SFA) and cholesterol levels, suggesting a decrease in atherogenicity^32^. Recent findings suggest that long non-coding RNAs (lncRNAs) can influence metabolic processes in response to shifting nutritional cues. Mounting evidence indicates that tissue-specific long non-coding RNAs (lncRNAs) play a functional role in response to nutritional changes. Certain lncRNAs play a role in coordinating physiological responses to nutrient deprivation, while others contribute to the pathogenesis of diseases influenced by nutrition^33^. Understanding how lncRNAs respond to changes in nutrition is crucial for comprehending the intricate downstream cascades triggered by dietary shifts. This knowledge can profoundly influence how we approach the treatment of metabolic diseases.

Despite our endeavors to understand the roles of lncRNAs MALAT1 and TUG1 in the context of metabolic disorders, the precise role of these lncRNAs in obesity and to understand how they interact with dietary factors remain unclear. In this study, we aimed to explore, the interaction between long non-coding RNA MALAT1 and TUG1 with dietary fatty acid quality indices concerning the visceral adiposity index (VAI) and body adiposity index (BAI) in overweight and obese women.

## Subjects and methods

### Study population

In this cross-sectional study, 346 healthy overweight and obese women were selected using community-based multi-stage simple random sampling after considering eligibility criteria. The participants were drawn from 20 randomly chosen health centers located throughout West and Central Tehran, Iran. The inclusion criteria comprised a body mass index (BMI) ranging from 25 to 40 kg/m^2^, and ages between 18 and 48 years. The criteria for not entering the study were individuals with a history of cardiovascular diseases (CVDs), kidney failure, stroke, thyroid disease, liver disease, cancer, inflammatory illnesses, and addiction to alcohol or drugs. Moreover, women undergoing menopause, pregnancy, and lactation, as well as those using weight-loss supplements, engaging in dieting within the previous year, and taking medications to lower blood pressure, glucose, and lipid levels in plasma, were not entering the study. All study participants provided written informed consent before the investigation commenced. The research received approval from the local ethics committee of Tehran University of Medical Sciences (TUMS) with the ethics number IR.TUMS.MEDICINE.REC.1401.073.

### Assessment of body composition

We applied a bioelectrical impedance analyzer (BIA) (Inbody 770 Co., Seoul, Korea) following the methods, safety precautions, and instructions outlined in the manufacturer's protocol^34^. Participants place their bare feet on the balance scale and grasp the BIA handles. The different readings were determined by the BIA using an electric signal that passes through the palms and soles of the feet. Before measuring, participants took off their shoes, excess clothes, and any metal items. It takes 15–20 s to check body composition, weight, trunk fat, body fat mass (BFM), visceral fat, fat-free mass index (FFMI), and fat mass index (FMI).

### Assessment of anthropometric indices

Before anthropometric measurements, participants were instructed to refrain from engaging in strenuous physical activity for 72 h and to fast for 12 h. While individuals were shoeless and wearing as minimal garments as possible, we utilized BIA 770 (South Korea) to determine body weight to the closest 100 g. A non-elastic tape was used to measure each participant’s height to the closest 0.5 cm while they were standing next to a wall in a typical stance. To determine the BMI (in square meters), we divided the weight (in kilograms) by the square of the height. According to the World Health Organization (WHO), obesity classes 1, 2, and 3 correspond to BMIs of 30–34.9%, 35–39.9%, and 40 kg/m^2^, respectively. A BMI of 25 to 29.9 kg/m^2^ is regarded as overweight. Without exerting any pressure on the body, we measured the waist circumference to the closest 0.5 cm using non-elastic tape at the place where the natural waistline ends. The waist-to-hip ratio (WHR) was estimated by dividing the waist circumference (WC) by the hip circumference (HC). To minimize measurement errors, all measures were performed by a single trained dietician.

### VAI and BAI assessment

BAI and VAI were calculated using the following formulas^14,35^

$${\text{BAI }} = { }\frac{{{\text{Hip Circumference }}\left( {{\text{cm}}} \right)}}{{height\, \left( m \right)^{1.5} }} - 18$$

$${\text{VAI}} = \left( {\frac{{{\text{Waist Circumference }}\left( {{\text{cm}}} \right)}}{{36.58 + \left( {1.89 \times BMI} \right)}}} \right) \times \left( {\frac{TG}{{0.81}}} \right) \times \left( {\frac{1.52}{{HDL}}} \right)$$

### Assessment of dietary intake

Habitual dietary intake frequency over the past 12 months was assessed using a 147-item semi-quantitative food frequency questionnaire (FFQ), whose validity and reliability have been approved^36^. Based on this questionnaire, the individuals were asked to indicate whether they consumed each food item on a daily, weekly, monthly, or yearly basis. After being told the typical size of each food item in the FFQ, participants were asked to rate the frequency of intake of each food item according to their standard unit on a daily, weekly, monthly, or yearly basis. The information from this questionnaire was entered into an Excel file that was made to determine the weight (in grams) of each food item. Items reported using standard units and home scales were converted to grams using the home scale conversion guide. So, for every item and every person, the equivalent of consumption was calculated. The dietary consumption data were converted and analyzed using the NUTRITIONIST 4 (N4) food analyzer from Hearst Corporation in San Bruno, California. Total energy, macronutrients, and micronutrients were determined using Hearst Corporation's Nutritionist 4 software in San Bruno, California^37^.

### Dietary fat quality indices

The FFQ was assessed to determine the meals to be included, which were then converted to grams per day using household measures. Cholesterol and saturated fatty acid levels were determined using the Iranian Food Composition Table (FCT) and N4 software. To evaluate the quality of fat, the Cholesterol-Saturated Fat Index (CSI) was employed. CSI provides data on the cholesterol and saturated fat content in various foods. The index is calculated by dividing cholesterol by the quantity of saturated fat in meal items, as assessed by the FFQ^38^. A low CSI indicates a lower quantity of saturated fat and/or cholesterol, suggesting that a diet with a lower CSI has a hypocholesterolemic and low atherogenic potential^31^.

$${\text{CSI}} = (1.01\; \times \;{\text{g saturated}}\;{\text{ fat}}) + (0.05\; \times \;{\text{mg}}\;{\text{cholesterol}}).$$

### Assessment of physical activity and other variables

The duration and frequency of regular daily activities over a week in the preceding year are included in The International Physical Activity Questionnaire (IPAQ). Metabolic equivalent hours (MET-h/week) are used to calculate the participants' weekly quantities of physical activity^39^. The reliability and validity of the International Physical Activity Questionnaire (IPAQ) have been previously assessed in Iranian adolescents^39^. A demographic questionnaire was used to collect data on demographic variables such as income and education status, income, and smoking status as well.

### Biochemical parameters assessment

Participants provided a morning blood sample between 8:00 and 10:00 AM following an overnight fasting period. The collected blood sample was then centrifuged, divided into smaller aliquots, and stored at − 80 °C. Subsequently, all samples underwent analysis using a standardized testing method. A glucose oxidase-phenol 4-aminoantipyrine peroxidase (GOD-PAP) test was used to determine fasting blood glucose (FBG). Triglycerides (TG) and total cholesterol (TC) were quantified using an enzyme endpoint called glycerol-3-phosphate oxidase-phenol 4-aminoantipyrine peroxidase (GPOPAP). The levels of low-density lipoprotein (LDL-c) and high-density lipoprotein (HDL-c) cholesterol were assessed using a direct enzymatic clearance test (Pars Azmoon Inc, Tehran, Iran). The minimum detectable level of insulin was 1.76 mIU/mL, with intra- and inter-class correlation coefficients of 2.11 and 4.4%, respectively. To determine the HOMA-IR, the homeostasis model assessment (HOMA) was computed as [(fasting plasma glucose fasting serum insulin)/22.5]^40^. At the School of Nutrition and Dietetics at TUMS, the Nutrition and Biochemistry Laboratory evaluated each sample using standardized procedures.

### Real-time quantitative polymerase chain reaction (PCR)

The concentration of the extracted RNA was assessed using NanoDrop at an absorbance of 260 nm. For optimal RNA purity, the A260/A280 ratio was maintained between 1.80 and 2.05, while the A260/A230 ratio fell within the range of 2.00–2.50. The integrity of the RNA was verified through agarose gel electrophoresis. For the subsequent step, the first-strand complementary DNA (cDNA) synthesis was conducted using 1000 ng of RNA that had undergone DNase treatment. This process utilized a cDNA synthesis kit from AnaCell, Tehran, Iran, following the manufacturer's instructions.

Quantitative real-time PCR was then carried out using the Step-One-Plus TM real-time PCR system by ABI Applied Biosystems, in conjunction with Bio FACTTM 2X Real-Time PCR Master Mix (for SYBR Green I), following the MIQE (Minimum Information for Publication of Quantitative Real-Time PCR Experiments) recommendations^41^. Following the real-time PCR, a melting curve analysis was conducted, involving a gradual temperature increase from 60 to 95 °C, followed by a return to 60 °C for 1 min. The SYBR Green fluorescence signal was continuously monitored, and the presence of single peaks, denoting a unique melting temperature (Tm), confirmed the success of the amplification process.

To assess gene alterations at the long non-coding RNA (lncRNA) level, we utilized the Schmittgen and Livak method, which calculates relative expression (2 − ΔCT) based on the ΔCT values obtained from real-time PCR. The reference gene used was 18s rRNA, and the expression of each sample was normalized to its corresponding reference gene value.

It is noteworthy that all samples exhibited Ct (Cycle threshold) values below 35. Calibration curves were employed to evaluate the amplification efficiency for both the target genes and reference genes, with the amplification efficiency (E) ranging from 95 to 100% for all genes. Since there was only one group under investigation (overweight and obese women), the 2-∆CT formula was applied to all measurements. For specific primer sequences used in the examination of MALAT1 and TUG1 gene expression, please refer to Table S1 for details.

#### Statistical analyses

Based on the cross-sectional study sample size calculation (β = 0.95, α = 0.05, power = 90%, effect size = 0.05, and number of predictive parameters = 7), a sample size of 346 has been determined^42^. The normality of quantitative variables was assessed using the Kolmogorov–Smirnov technique, and the histogram curve was evaluated (*P* > 0.05). Mean and standard deviation (SD) were used to report quantitative and number (percent) for categorical variables. Participants’ characteristics among tertiles of transcript levels of MALAT1 TUG1 and CSI were analyzed using one-way analysis of variance (ANOVA) for continuous variables and chi-squared for categorical variables. Analysis of covariance (ANCOVA) was performed for adjustment models. All associations were reported in the crude model and after adjustment for age, BMI, energy intake, and physical activity. Generalized linear models (GLMs) as linear regression were exerted to analyze the interaction between mRNA expression of MALAT1, TUG1, and CSI on VAI and BAI indexes in the crude and adjusted model. The adjustment was applied based on age, energy intake, smoking, income, and physical activity in Model 1. Analysis output was reported as β, standard error (SE) and 95% confidence interval (CI). SPSS version 26.0 (SPSS, Chicago, IL, USA) was used for all statistical analyses. All published *P*-values were two-sided, with a significance level set at less than 0.05 to be considered statistically significant. *P*-values less than 0.1 were deemed marginally significant.

### Ethical approval and consent to participate

The study protocol has been approved by the ethics committee of Tehran University of Medical Sciences (TUMS) with the following identification: IR.TUMS.MEDICINE.REC.1401.073. Each participant was completely informed about the study protocol and provided a written and informed consent form before taking part in the study. All methods were carried out under relevant guidelines and regulations or the declaration of Helsinki.

## Results

### Study population characteristics

The statistical analysis included a total of 346 women.The transcript levels of MALAT1, TUG1, and CSI mean and standard deviation (SD) in this study were 1.520 (6.010), 1.700 (6.024), and 13.244 (5.714), respectively. Individuals’ average age, weight, and BMI were 36.568 ± 8.978 years, 80.762 ± 10.518 kg, and 31.215 ± 4.182 kg/m^2^ respectively. The mean (SD) of anthropometric and body composition such as BFM, WC, and WHR were 33.990 (8.085), 99.533 (9.993), and 1.159 (4.486) respectively.

### General characteristics of participants among MALAT1, TUG1 mRNA expression, and CSI tertiles

Table 1 shows the characteristics of the participants, their socioeconomic status, and anthropometric and biochemical assessments before and after adjustment for cofounders. In the crude model and after adjustments for age, physical activity, BMI, and total energy consumption, there were no statistically significant mean differences observed for social characteristics, anthropometrics, biochemical assessment, and categorical variables between MALAT1 and TUG1 expression tertiles. However, there was a marginally significant mean difference for total cholesterol among tertiles of MALAT1 transcript levels before adjustment (*p* = 0.056), and after controlling for confounders, it remained significant (*p* = 0.046).

**Table 1:** Participants' characteristics among tertiles of MALAT1 and TUG1 transcript levels and CSI in obese and overweight women (n = 346).

Variables	MALAT1 tertiles			*p*-value	*P*-value*	TUG1 tertiles			*p*-value	*P*-value*
	T1	T2	T3			T1	T2	T3		
	< 0.035	0.036–0.309	> 0.310			< 0.053	0.053–0.264	> 0.265		
	N = 115	N = 116	N = 115			N = 117	N = 118	N = 118		
	Mean ± SD					Mean ± SD				
Age (year)	35.657 ± 9.178	37.078 ± 9.383	36.751 ± 8.936	0.472	0.355	35.926 ± 9.528	36.589 ± 8.576	37.313 ± 9.077	0.505	0.283
PA (MET)	985.352 ± 1223.620	828.232 ± 969.264	1153.323 ± 1096.564	0.120	0.121	847.426 ± 914.507	965.345 ± 1159.972	1067.898 ± 1102.361	0.452	0.366
Weight (kg)	80.281 ± 14.171	82.394 ± 11.543	80.312 ± 13.144	0.367	0.852	79.725 ± 13.244	82.328 ± 12.709	81.324 ± 12.585	0.295	0.536
Height (cm)	161.450 ± 5.704	161.478 ± 5.602	161.256 ± 5.886	0.950	0.994	161.425 ± 5.656	161.474 ± 5.873	160.800 ± 5.994	0.615	0.514
BMI (kg/m^2^)	31.054 ± 4.115	31.803 ± 4.240	30.844 ± 4.261	0.192	0.437	30.951 ± 4.017	31.667 ± 4.364	31.423 ± 4.337	0.423	0.602
WC (cm)	99.531 ± 10.081	100.845 ± 9.726	98.501 ± 10.878	0.223	0.786	98.901 ± 9.812	100.901 ± 10.890	99.397 ± 9.734	0.294	0.189
WHR	0.938 ± 0.052	1.722 ± 8.398	0.929 ± 0.0548	0.367	0.556	0.933 ± 0.055	1.703 ± 8.290	0.933 ± 0.047	0.368	0.263
FM(kg)	33.527 ± 7.903	35.547 ± 8.453	33.441 ± 8.201	0.091	0.394	33.597 ± 8.268	35.135 ± 8.589	33.637 ± 7.756	0.264	0.251
FFM(kg)	46.447 ± 6.167	46.527 ± 5.847	46.180 ± 6.578	0.907	0.688	46.439 ± 5.470	46.778 ± 6.351	46.379 ± 5.771	0.854	0.727
BF%	41.864 ± 5.103	53.553 ± 116.558	41.960 ± 5.263	0.325	0.790	41.988 ± 5.445	53.060 ± 115.089	42.291 ± 5.151	0.356	0.982
FMI	13.203 ± 3.210	13.765 ± 3.381	13.226 ± 3.380	0.352	0.482	13.205 ± 3.298	13.649 ± 3.398	13.540 ± 3.413	0.581	0.956
FFMI	17.919 ± 1.477	17.903 ± 1.538	18.896 ± 12.343	0.494	0.526	17.809 ± 1.518	17.914 ± 1.556	17.935 ± 1.556	0.802	0.764
Obesity degree%	143.582 ± 22.022	145.512 ± 23.751	143.628 ± 20.337	0.753	0.663	144.298 ± 19.340	145.117 ± 23.914	144.962 ± 22.999	0.957	0.474
Biochemical parameters										
FBG (mg/l)	86.341 ± 7.239	87.606 ± 12.084	87.000 ± 7.896	0.710	0.788	86.355 ± 7.853	86.432 ± 7.084	87.759 ± 12.005	0.590	0.743
Insulin	1.248 ± 0.236	1.201 ± 0.260	1.217 ± 0.213	0.491	0.273	1.202 ± 0.215	1.247 ± 0.225	1.229 ± 0.252	0.559	0.501
HOMA index	3.293 ± 1.112	3.254 ± 1.351	3.371 ± 1.409	0.869	0.589	3.204 ± 1.219	3.320 ± 1.289	3.393 ± 1.330	0.698	0.940
TG (mg/l)	124.189 ± 65.902	112.651 ± 64.318	123.214 ± 74.767	0.548	0.483	116.033 ± 65.437	122.648 ± 58.155	122.084 ± 68.823	0.813	0.701
T-Chol (mg/l)	180.515 ± 20.442	180.515 ± 29.383	192.942 ± 35.350	**0.056**	**0.049**	183.186 ± 39.279	181.918 ± 37.374	189.445 ± 32.179	0.379	0.661
LDL-c (mg/L)	95.227 ± 26.227	91.515 ± 20.442	97.685 ± 24.166	0.318	0.333	91.152 ± 23.565	94.013 ± 25.065	99.024 ± 24.455	0.150	0.308
HDL-c (mg/L)	46.278 ± 10.626	45.924 ± 9.853	47.557 ± 12.347	0.655	0.927	45.661 ± 10.040	46.121 ± 10.991	10.126 ± 1.111	0.703	0.874
CHOL.HDL	3.947 ± 0.885	4.116 ± 1.136	4.430 ± 2.198	0.158	0.110	4.284 ± 2.265	4.057 ± 1.026	4.183 ± 1.192	0.698	0.723
Categorical variables										
	N (%)					N (%)				
Education status				0.438	0.172				0.294	0.570
Illiterate	2 (50)	1 (25)	1 (25)			1 (25)	1 (25)	2 (50)		
Preschool	4 (44.4)	2 (22.2)	3 (33.3)			2 (18.2)	2 (18.2)	7 (63.6)		
Intermediate	4 (18.2)	7 (31.8)	11 (50)			6 (25)	12 (50)	6 (25)		
High school	1 (14.3)	4 (57.1)	2 (28.6)			2 (25)	1 (12.5)	5 (62.5)		
Diploma	31 (29.5)	36 (34.3)	38 (36.2)			37 (33.3)	40 (36)	34 (30.6)		
Upper diploma	6 (23.1)	12 (46.2)	8 (30.8)			9 (37.5)	4 (16.7)	11 (45.8)		
Bachelor and higher	65 (39.4)	53 (32.1)	47 (28.5)			57 (34.8)	56 (34.1)	51 (31.1)		
Income (Rial)				0.394	0.520				0.36	0.279
< 5,000,000	1 (20)	1(20)	3(60)			0 (0)	5 (100)	0 (0)		
5,000,000 to 10,000,000	3 (25)	4 (33.3)	5 (41.7)			3 (27.3)	3 (27.3)	5 (45.5)		
10,000,000 to 15,000,000	32 (36)	36 (40.4)	21 (23.6)			31 (34.4)	25 (27.8)	34 (37.8)		
> 15,000,000	59 (34.7)	53 (31.2)	58 (34.1)			64 (36.2)	62 (35)	51 (28.8)		
Smoking				0.193	**0.084**				0.517	0.322
Yes	5 (20.8)	12 (50)	7 (29.2)			9 (40.9)	5(22.7)	8 (36.4)		
No	105 (34.8)	99 (32.8)	98 (32.5)							

Variables	CSI tertiles			*p*-value	*P*-value*					
	T1	T2	T3							
	< 9.91	9.92–14.57	> 14.60							
	N = 126	N = 126	N = 126							
	Mean ± SD									
Age (year)	37.664 ± 8.680	37.349 ± 9.229	34.817 ± 9.199	0.024	0.451					
PA (MET)	839.327 ± 1027.524	1092.786 ± 1205.068	1048.355 ± 1046.475	0.181	0.374					
Weight (kg)	79.792 ± 10.581	80.127 ± 11.204	81.953 ± 12.106	0.265	0.888					
Height (cm)	160.686 ± 5.868	161.277 ± 5.865	161.643 ± 5.635	0.417	0.968					
BMI (kg/m^2^)	30.956 ± 3.705	30.762 ± 3.777	31.330 ± 4.075	0.495	0.564					
WC (cm)	98.626 ± 9.093	98.733 ± 9.528	100.199 ± 10.096	0.348	0.857					
WHR	0.932 ± .050	0.934 ± .054	0.939 ± .054	0.555	0.796					
FM(kg)	33.881 ± 7.792	33.591 ± 7.606	35.326 ± 8.557	0.184	0.602					
FFM(kg)	46.062 ± 5.349	46.483 ± 5.483	46.537 ± 5.779	0.758	0.585					
BF%	42.034 ± 5.278	41.608 ± 4.950	42.489 ± 5.808	0.427	0.657					
FMI	13.258 ± 3.122	12.968 ± 2.940	13.552 ± 3.256	0.331	0.290					
FFMI	17.820 ± 1.481	18.885 ± 11.709	17.774 ± 1.541	0.351	0.331					
Obesity degree%	144.168 ± 17.937	141.408 ± 21.089	145.675 ± 19.228	0.212	0.100					
Biochemical parameters										
FBG (mg/l)	87.988 ± 10.607	87.146 ± 9.705	86.419 ± 8.235	0.621	0.992					
Insulin	1.196 ± 0.237	1.221 ± 0.218	1.226 ± 0.231	0.684	0.580					
HOMA index	3.422 ± 1.403	3.170 ± 1.171	3.483 ± 1.271	0.269	0.422					
TG (mg/l)	123.690 ± 81.127	124.067 ± 70.696	113.435 ± 49.645	0.602	0.701					
T-Chol (mg/l)	184.750 ± 31.244	184.225 ± 39.719	181.565 ± 36.173	0.857	0.661					
LDL-c (mg/L)	95.083 ± 23.117	92.843 ± 25.158	95.161 ± 23.154	0.779	0.308					
HDL-c (mg/L)	47.405 ± 10.411	46.427 ± 12.131	46.097 ± 8.423	0.732	0.874					
CHOL.HDL	4.077 ± 1.185	4.277 ± 2.017	4.024 ± 0.937	0.536	0.515					
Categorical variables										
	N (%)									
Education status				0.166	**0.007**					
Illiterate	3 (75)	1 (25)	0 (0)							
Preschool	6 (46.2)	4 (30.8)	3 (23.1)							
Intermediate	13 (52)	6 (24)	6 (24)							
High school	4 (50)	1 (12.5)	3 (37.5)							
Diploma	36 (30.8)	45 (38.5)	36 (30.8)							
Upper diploma	12 (44.4)	9 (33.3)	6 (22.2)							
Bachelor and higher	51 (28)	59 (32.4)	72 (39.6)							
Income (Rial)				**0.058**	**0.069**					
< 5,000,000	2 (33.3)	4 (66.7)	0 (0)							
5,000,000 to 10,000,000	8 (57.1)	2 (14.3)	4 (28.6)							
10,000,000 to 15,000,000	41(38)	37 (34.3)	30 (27.8)							
> 15,000,000	59 (31.4)	55 (29.3)	74 (39.4)							
Smoking				**0.002**	**0.009**					
Yes	5 (19.2)	4 (15.4)	17(65.4)							
No	119 (34)	122 (34.9)	109 (31.1)							

Significant and marginally significant values are in [bold].

BMI, Body mass index, FM: Fat mass; FFM, fat-free mass; BF%, body fat%; FFMI, fat-free mass index; FMI, fat mass index; BAI, body composition index; VAI, visceral adiposity index; TG, triglyceride; T-Chol, total cholesterol, FBG, fasting blood glucose; HOMA-IR, Homeostatic model assessment-insulin resistance; WC, waist circumference; WHR, waist to hip ratio, HDL-c, high-density lipoprotein cholesterol; LDL, low-density lipoprotein cholesterol; CHOL.HDL, total cholesterol to high-density lipoprotein cholesterol ratio; PA, physical activity; MET, metabolic equivalents; TUG1, taurine up-regulated 1, MALAT1, metastasis associated lung adenocarcinoma transcript 1; CSI, cholesterol-saturated fat index; N, number.

*P*-values resulted from a one-way analysis of variance (ANOVA) test and were reported as mean ± standard deviation (SD).

*P*-values* resulted from the analysis of the covariance (ANCOVA) test. Variables adjust for age (year), BMI (kg/m^2^), energy intake (kcal), and physical activity (MET). BMI is considered the collinear variable for anthropometric and body composition variables.

A chi-square test for categorical variables has been done. Categorical variables are present by N (%).

*P*-value < 0.05 was considered significant and *p*-values 0.05 and 0.10 were considered as marginally significant.

Furthermore, there was a significant mean difference among tertiles of CSI for education status (*p* = 0.007) and smoking (*p* = 0.009), and a marginally significant mean difference for income (*p* = 0.069) after controlling confounders.

### The difference in means of BAI and VAI among tertiles of MALAT1 and TUG1 transcript levels and CSI in obese and overweight women

There was no significant mean difference among the tertiles of MALAT1, TUG1 transcript levels, and CSI for VAI and BAI (*p* > 0.05) (Table 2).

**Table 2:** The difference means of BAI and VAI among tertiles of MALAT1 and TUG1 transcript levels and CSI in obese and overweight women (n = 346).

Variables	MALAT1 tertiles			*p*-value	*P*-value*	TUG1 tertiles			*p*-value	*P*-value*
	T1	T2	T3			T1	T2	T3		
	< 0.035	0.036–0.309	> 0.310			< 0.053	0.053–0.264	> 0.265		
	N = 115	N = 116	N = 115			N = 117	N = 118	N = 118		
	Mean ± SD					Mean ± SD				
BAI	55.621 ± 3.958	54.710 ± 4.560	54.993 ± 3.894	0.550	0.637	55.060 ± 4.602	55.422 ± 4.501	55.416 ± 3.990	0.911	0.646
VAI	93.550 ± 58.045	90.408 ± 66.050	106.129 ± 107.938	0.484	0.346	92.994 ± 63.627	95.497 ± 57.619	96.649 ± 72.959	0.949	0.952

Variables	CSI tertiles			*p*-value	*P*-value*					
	T1	T2	T3							
	< 9.91	9.92–14.57	> 14.60							
	N = 126	N = 126	N = 126							
	Mean ± SD									
BAI	55.309 ± 3.595	55.072 ± 4.809	55.374 ± 4.568	0.930	0.246					
VAI	101.977 ± 93.840	102.401 ± 94.220	87.470 ± 45.916	0.492	0.337					

BAI, body composition index; VAI, visceral adiposity index; TUG1, taurine up-regulated 1; MALAT1, metastasis associated lung adenocarcinoma transcript 1, CSI, cholesterol-saturated fat index.

*P*-values resulted from a one-way analysis of variance (ANOVA) test and were reported as mean ± standard deviation (SD).

*P*-values* resulted from the analysis of the covariance (ANCOVA) test. Variables adjust for age (year), BMI (kg/m^2^), energy intake (kcal), and physical activity (MET). BMI is considered the collinear variable for anthropometric and body composition variables.

A chi-square test for categorical variables has been done. Categorical variables are present by N (%).

*P*-value < 0.05 was considered significant and *p*-values 0.05 and 0.10 were considered marginally significant.

### Dietary intake of study population over MALAT1 and TUG1 expression tertiles

Table 3 shows the participants' intake of specific nutrients and food groups across MALAT1 and TUG1 expression tertiles. After controlling energy intake, lower intakes of carbohydrates (*p* = 0.038), fat (*p* = 0.019), oleic acid (*p* = 0.037), vitamin B5 (*p* = 0.040), and legumes (*p* = 0.031) were found in participants in the highest tertile of MALAT 1 transcript level. Meanwhile, linolenic acid (*p* = 0.095), vitamin A (*p* = 0.064), vitamin B1 (*p* = 0.086), and poultry (*p* = 0.073) intake were marginally significant mean differences among tertiles of MALAT 1 mRNA expression. Participants with lower mRNA expression of TUG1 showed a significantly lower intake of fruits (*p* = 0.007), and dietary vitamin K (*p* = 0.026) was lower in the top tertiles of the TUG1 transcript level. Additionally, there was a marginally significant difference in omega-3 intake (*p* = 0.071), vitamin B1 (*p* = 0.079), and high-fat dairy intake (*p* = 0.058).

**Table 3:** Dietary intake among tertiles of MALAT1 and TUG1 transcript level in obese and overweight women (n = 346).

Variables	MALAT1 tertiles			*P*-value	*P*-value*	TUG1 tertiles			*p*-value	*P*-value*	CSI tertiles			*p*-value	*P*-value*
	T1	T2	T3			T1	T2	T3			T1	T2	T3		
	< 0.035	0.036–0.309	> 0.310			< 0.053	0.053–0.264	> 0.265							
	N = 115	N = 116	N = 115			N = 117	N = 118	N = 118			T_1_ (n = 99	T_2_ (n = 104)	T_3_ (n = 76)		
	Mean ± SD					Mean ± SD									
Energy intake (kcal/d)	2602.146 ± 854.611	2729.974 ± 756.472	2587.128 ± 802.058	0.355	–	2616.285 ± 847.318	2673.289 ± 740.753	2606.203 ± 85.772	0.798	–	2136.53 ± 601.19	2650.61 ± 674.27	3151.67 ± 612.17		–
Carbohydrate (g/d)	372.637 ± 129.305	378.590 ± 114.451	371.968 ± 127.365	0.910	**0.038**	366.497 ± 127.078	377.799 ± 116.854	372.531 ± 122.925	0.789	0.471	66.53 ± 17.27	91.41 ± 20.92	112.36 ± 28.38		** < 0.001**
Protein (g/d)	91.509 ± 32.165	93.517 ± 32.103	90.450 ± 29.994	0.766	0.651	92.804 ± 33.160	93.688 ± 32.401	89.847 ± 29.993	0.647	0.541	305.72 ± 102.04	385.51 ± 120.18	435.97 ± 96.75		** < 0.001**
Fat (g/d)	91.461 ± 35.289	102.264 ± 35.531	90.899 ± 32.621	**0.024**	**0.019**	95.301 ± 37.075	96.456 ± 33.277	92.361 ± 33.638	0.667	0.596	78.52 ± 29.67	91.87 ± 26.72	116.48 ± 30.52		**0.03**
Oleic acid	27.668 ± 12.024	31.339 ± 12.539	27.173 ± 10.678	0.18	**0.037**	29.656 ± 13.380	28.965 ± 11.329	27.349 ± 10.636	0.335	0.147	24.86 ± 11.50	26.86 ± 8.71	33.18 ± 9.97		0.06
Linoleic acid(g/d)	16.378 ± 8.629	18.777 ± 9.008	16.739 ± 8.333	**0.089**	0.227	17.840 ± 10.001	17.392 ± 8.132	16.271 ± 7.064	0.371	0.284	16.85 ± 9.56	16.76 ± 7.10	18.13 ± 7.14		** < 0.001**
Linolenic acid(g/d)	1.112 ± 0.565	1.329 ± 0.701	1.175 ± 0.676	**0.041**	**0.095**	1.195 ± 0.686	1.254 ± 0.654	1.150 ± 0.584	0.485	0.591	1.03 ± 0.66	1.19 ± 0.54	1.50 ± 0.61		0.46
Eicosapentaenoic acid (g/d)	.032 ± .038	.030 ± .039	.027 ± .031	0.708	0.715	.027 ± .040	.031 ± .034	.031 ± .041	0.584	0.595	0.01 ± 0.02	0.03 ± 0.03	0.04 ± 0.04		** < 0.001**
Docosahexaenoic acid (g/d)	.104 ± .115	.100 ± .121	.090 ± .097	0.636	0.657	.089 ± .120	.102 ± .108	.104 ± .125	0.598	0.606	0.06 ± 0.06	0.11 ± 0.12	0.14 ± 0.13		** < 0.001**
Omega3(g/d)	1.243 ± .561	1.524 ± .721	1.327 ± .698	**0.023**	0.161	1.254 ± 0.678	1.537 ± 0.694	1.280 ± 0.638	**0.013**	**0.071**					
Omega6(g/d)	15.815 ± 7.390	18.873 ± 8.665	16.736 ± 8.037	**0.048**	0.243	16.726 ± 8.966	18.287 ± 8.134	16.091 ± 7.034	0.182	0.470					
CSI	12.686 ± 5.203	14.209 ± 6.556	12.733 ± 5.120	0.080	0.214	13.348 ± 5.848	13.557 ± 5.603	13.015 ± 5.676	0.777	0.877					
Vitamins															
Vitamin A (μg/day)	827.813 ± 487.105	752.772 ± 388.081	725.797 ± 335.669	0.169	**0.064**	766.695 ± 426.826	785.934 ± 412.431	722.568 ± 361.942	0.488	0.558					
Vitamin D(μg/day)	2.113 ± 1.599	1.946 ± 1.595	1.775 ± 1.354	0.269	0.227	2.031 ± 1.615	2.064 ± 1.743	1.770 ± 1.318	0.317	0.350					
Vitamin E (mg/day)	16.890 ± 9.654	17.983 ± 9.295	15.943 ± 7.347	0.238	0.444	17.323 ± 9.241	17.184 ± 9.253	15.969 ± 7.471	0.449	0.457					
Vitamin K(mg/day)	326.382 ± 394.962	281.308 ± 257.702	281.534 ± 252.885	0.467	0.341	358.725 ± 398.292	274.180 ± 243.778	260.327 ± 255.817	**0.038**	**0.026**					
Vitamin B1(mg/day)	2.168 ± 0.785	2.162 ± 0.664	2.138 ± 0.718	0.951	**0.086**	2.178 ± 0.814	2.129 ± 0.656	2.178 ± 0.741	0.851	**0.079**					
Vitamin B2(mg/day)	2.326 ± 0.914	2.313 ± 0.886	2.248 ± 0.812	0.780	0.348	2.345 ± 0.943	2.298 ± 0.788	2.209 ± 0.813	0.486	0.211					
Vitamin B3(mg/day)	26.170 ± 10.672	27.192 ± 10.588	26.258 ± 9.273	0.714	0.854	27.343 ± 11.564	26.162 ± 10.211	26.382 ± 9.125	0.666	0.119					
Vitamin B5 (mg/day)	6.764 ± 3.047	6.449 ± 1.947	6.337 ± 2.142	0.411	**0.040**	6.676 ± 3.080	6.597 ± 1.973	6.231 ± 2.109	0.355	0.191					
Vitamin B6 (mg/day)	2.212 ± 0.778	2.247 ± 0.766	2.195 ± 0.754	0.880	0.534	2.248 ± 0.804	2.225 ± 0.751	2.159 ± 0.736	0.669	0.363					
Vitamin B9 (μg/day)	712.028 ± 254.965	700.400 ± 229.644	694.195 ± 221.841	0.855	0.129	705.977 ± 255.521	699.482 ± 215.701	708.952 ±	0.954	0.427					
Vitamin B12(μg/day)	4.586 ± 2.672	4.193 ± 2.154	4.153 ± 2.152	0.325	0.104	4.349 ± 2.332	4.476 ± 2.802	4.122 ± 1.817	0.529	0.612					
Vitamin C (mg/day)	184.227 ± 93.514	203.590 ± 144.119	186.766 ± 116.727	0.433	0.766	177.174 ± 92.955	198.530 ± 107.503	175.324 ± 106.273	0.175	0.206					
Food groups															
Refined grains (g/d)	462.952 ± 210.358	504.352 ± 276.564	456.405 ± 222.096	0.394	0.959	474.331 ± 232.837	486.361 ± 277.966	477.577 ± 190.971	0.947	0.704	331.80 ± 219.09	379.17 ± 191.55	397.52 ± 219.72		**0.01**
Whole grains (g/d)	65.780 ± 60.599	77.167 ± 73.844	76.463 ± 74.180	0.406	0.456	79.115 ± 99.963	72.368 ± 65.946	72.593 ± 65.938	0.771	0.687	53.24 ± 47.75	61.59 ± 54.19	77.42 ± 73.40		0.82
Total fiber (g/d)	48.369 ± 24.381	47.127 ± 19.355	48.253 ± 20.461	0.894	0.191	48.277 ± 24.505	46.719 ± 18.358	48.739 ± 22.562	0.774	0.321					
Vegetables(g/d)	401.449 ± 247.507	371.634 ± 241.267	371.246 ± 246.437	0.583	0.416	362.508 ± 223.788	396.610 ± 244.876	370.068 ± 248.130	0.537	0.625	288.84 ± 183.46	424.89 ± 241.68	445.77 ± 262.62		**0.003**
Fruits(g/d)	422.511 ± 243.382	503.688 ± 402.483	428.339 ± 291.393	0.113	0.253	385.085 ± 269.311	502.820 ± 361.756	413.485 ± 267.387	**0.011**	**0.007**	388.93 ± 312.69	510.63 ± 334.57	648.63 ± 34.94		0.92
Low fat dairy(g/d)	314.256 ± 214.851	277.691 ± 234.791	285.682 ± 202.195	0.430	0.269	283.819 ± 198.639	314.929 ± 254.523	282.858 ± 193.719	0.460	0.546					
High fat dairy(g/d)	85.897 ± 125.843	115.513 ± 171.192	74.736 ± 114.151	0.086	0.176	109.118 ± 175.067	105.271 ± 145.066	67.035 ± 102.916	**0.061**	**0.058**					
White meat(g/d)	40.981 ± 33.034	54.027 ± 62.170	45.692 ± 30.810	**0.092**	0.164	45.873 ± 41.948	49.213 ± 59.606	47.519 ± 32.217	0.864	0.915					
Red meat(g/d)	23.683 ± 21.499	22.075 ± 18.086	20.628 ± 23.714	0.575	0.464	22.138 ± 19.291	24.107 ± 25.643	19.725 ± 17.650	0.308	0.369					
Organ meat(g/d)	2.419 ± 3.419	2.452 ± 3.805	1.806 ± 2.440	0.272	0.337	1.921 ± 2.664	2.292 ± 3.230	2.382 ± 4.051	0.558	0.559					
Processed meat(g/d)	5.417 ± 11.060	6.929 ± 10.774	6.760 ± 17.146	0.655	0.714	6.647 ± 14.083	4.970 ± 10.169	6.804 ± 14.177	0.506	0.388					
Fish (g/d)	11.568 ± 11.327	11.375 ± 12.873	9.447 ± 9.428	0.321	0.365	10.153 ± 11.195	10.817 ± 12.423	11.507 ± 12.450	0.707	0.689	7.06 ± 6.33	12.59 ± 12.24	15.62 ± 15.97		** < 0.001**
Poultry(g/d)	29.413 ± 26.025	42.651 ± 54.853	36.245 ± 27.560	**0.042**	**0.073**	35.719 ± 36.869	38.396 ± 51.701	36.012 ± 28.869	0.864	0.931	23.03 ± 18.74	35.25 ± 26.63	50.75 ± 62.74		**0.002**
Legume(g/d)	54.065 ± 50.175	42.513 ± 36.350	41.552 ± 28.896	**0.036**	**0.031**	40.463 ± 32.868	51.084 ± 45.345	46.099 ± 38.808	0.133	0.161	42.47 ± 34.57	51.43 ± 42.42	46.40 ± 42.31		0.27
Nuts(g/d)	13.298 ± 15.012	17.239 ± 22.343	15.789 ± 18.661	0.300	0.423	16.048 ± 19.002	17.143 ± 23.305	12.541 ± 15.259	0.190	0.213	9.45 ± 10.99	14.59 ± 15.63	21.40 ± 20.64		0.25
Olive (g/d)	5.529 ± 6.904	5.021 ± 6.518	4.855 ± 8.542	0.783	0.760	5.171 ± 8.583	6.016 ± 7.235	3.944 ± 5.812	0.109	0.121					
Egg (g/d)	22.059 ± 13.145	26.560 ± 19.500	25.339 ± 22.317	0.186	0.236	24.769 ± 17.506	26.118 ± 21.561	23.667 ± 16.188	0.619	0.680	12.60 ± 7.02	21.47 ± 9.40	33.85 ± 17.9		** < 0.001**

Significant and marginally significant values are in [bold].

T, tertile; MALAT1. metastasis associated lung adenocarcinoma transcript 1; TUG1, taurine up-regulated 1; CSI, cholesterol-saturated fat index.

*P*-values resulted from a one-way analysis of variance (ANOVA) test and were reported as mean ± standard deviation (SD).

*P*-values* resulted from the analysis of covariance (ANCOVA) test. Variables adjust for energy intake (kcal).

*P*-value < 0.05 was considered as significant and *p*-value 0.05 and 0.10 were considered as marginally significant.

### The association of MALAT1 and TUG1 mRNA expression with BAI and VAI

The association between MALAT1 and TUG1 mRNA expression and BAI and VAI was presented in Table 4. The crude model revealed a significant positive relationship between the transcript level of MALAT1 and VAI (β = 3.646, 95%CI 1.950–5.341, *p* < 0.001) (Table 4). After adjusting for confounding factors such as age, BMI, energy intake, and physical activity, a significant positive association persisted between MALAT1 transcript level and VAI (β = 8.338, 95%CI 6.110 to 10.566, *p* < 0.001). However, no significant association of the mRNA level of TUG1 with BAI and VAI was observed in both the crude and adjusted models **(**Table 4**).**

**Table 4:** Association of MALAT1 and TUG1 transcript levels with BAI and VAI in obese and overweight women (n = 346).

Risk factors	Models	β	SE	95%CI Lower, upper	*P*-value
MALAT1					
BAI	Crude	− 0.029	0.076	− 0.179 to 0.121	0.706
	Model 1	− 0.046	0.049	− 0.143 to 0.051	0.353
VAI	Crude	3.646	0.860	1.950 to 5.341	**< 0.001**
	Model 1	8.338	1.129	6.110 to 10.566	**< 0.001**
TUG1					
BAI	Crude	− 0.030	0.058	− 0.144 to 0.085	0.610
	Model 1	0.010	0.035	− 0.058 to 0.079	0.765
VAI	Crude	− 0.481	0.594	− 1.651 to 0.690	0.419
	Model 1	− 0.437	0.612	− 1.644 to 0.771	0.476

Significant values are in [bold].

^†^Linear regression; CI: confidence interval; SE: standard error; MALAT1: Metastasis Associated Lung Adenocarcinoma Transcript 1, TUG1: Taurine Up-Regulated 1; BAI: body composition index, VAI: visceral adiposity index.

Model 1: Adjusted for age, PA, BMI, and energy intake.

*P*-value < 0.05 was considered as significant and *p*-value 0.05 and 0.10 were considered as marginally significant.

### The interaction between MALAT1 and TUG1 mRNA expression and CSI on BAI and VAI

The interaction between MALAT1 expression and CSI on BAI and VAI in the crude model and after adjustment for age, energy intake, smoking, income, and physical activity in model 1 is presented in Table 5. In the crude model, a significant positive interaction was observed between MALAT1 expression and CSI on VAI (β: 1.287, 95%CI 0.806, 1.769, *p* < 0.001), and a marginally significant positive interaction was noted between MALAT1 expression and CSI on BAI (β: 0.105, 95%CI − 0.015, 0.226, *p* = 0.088). After controlling for confounding variables in model 1, a significant positive interaction between MALAT1 expression and CSI was observed on BAI (β: 0.130, 95%CI 0.019, 0.240, *p* = 0.022), and a marginal positive interaction was seen on VAI (β: 0.718, 95%CI − 0.028, 1.463, *p* = 0.059). There was no significant interaction between CSI and TUG1 expression on BAI and VAI in both crude and adjustment models.

**Table 5:** Interaction of MALAT1 and TUG1 transcript level with the CSI in relation to BAI and VAI among women categorized as overweight or obese. (n = 346).

Risk factors	Interaction Models	Β	SE	95%CI Lower, upper	*P*-value
MALAT1					
BAI	Crude	0.105	0.061	-0.015 to 0.226	**0.088**
	Model 1	0.130	0.056	0.019 to 0.240	**0.022**
VAI	Crude	1.287	0.245	0.806 to 1.76	** < 0.001**
	Model 1	0.718	0.380	-0.028 to 1.463	**0.059**
TUG1					
BAI	Crude	-0.018	0.025	-0.068 to 0.033	0.498
	Model 1	-0.036	0.023	-0.081 to 0.010	0.124
VAI	Crude	0.089	0.135	-0.177 to 0.355	0.513
	Model 1	0.128	0.172	-0.210 to 0.465	0.458

Significant and marginally significant values are in [bold].

β: beta, CSI: cholesterol-saturated fat index, CI: confidence interval, SE: standard error, MALAT1: Metastasis Associated Lung Adenocarcinoma Transcript 1, TUG1: Taurine Up-Regulated 1, BAI: Body composition index, VAI: visceral adiposity index.

The generalized linear model was performed to identify the interaction between MALAT1 and TUG1 with CSI tertiles on BAI and VAI.

Adjusted Model 1: was adjusted for age, energy intake, physical activity, income, and smoking.

*P*-value < 0.05 was considered as significant and *p*-value 0.05 and 0.10 were considered as marginally significant.

## Discussion

To the best of our knowledge, this is the first study investigating the interactions between mRNA expression of lncRNAs, MALAT1, and TUG1, with dietary fat quality index (CSI) on VAI and BAI in overweight and obese women. Our study can provide evidence regarding positive interactions between increased CSI and MALAT1 expression on VAI and BAI. However, no substantially significant interactions between TUG1 and CSI were identified in terms of the above-mentioned markers.

VAI, a novel sex-specific index, is focused on BMI, WC, TG, and HDL-c.

Compared to the effect of a single factor, VAI provides more comprehensive estimates of overall variables, thus better identifying visceral adiposity dysfunction associated with cardio-metabolic risk factors^43^. BAI is a useful established indicator of % fat in both men and women, and it has also been considered to serve as a better predictor of health outcomes than BMI itself^44^. Nevertheless, the interaction of these two novel indicators of obesity has been scarcely investigated in the context of lncRNAs^45,46^. The possible role of lncRNAs in pathogenesis and underlying mechanisms of obesity and related abnormalities have recently started to emerge^47,48^. However, no previous research has been conducted directly on lncRNAs-CSI interaction and lncRNAs-VAI/BAI, and even less has looked at the association of these molecules pertinent to metabolic disorders^49,50^; therefore, our results shed light on an unknown relationship between MALAT1 and TUG1 expression with CSI on VAI and BAI. A recent study among patients with atherosclerosis reported the involvement of MALAT1 in cholesterol accumulation in oxidized low-density lipoprotein (ox-LDL)-stimulated macrophages^51^. Jia et al. also demonstrated that MALAT1 transcript level was statistically upregulated in cardiomyocytes by saturated fatty acids (SFAs) during palmitic acid-induced hepatic steatosis treatment^52^. The contribution of lncRNAs in obesity-related research has been mostly performed in vitro and animal model study more than in human tissues^53–55^ and several lncRNAs were mapped to genetic loci that influence fat deposition regulation and lipid homeostasis^49,56,57^. Among these, increased expression of MALAT1 in pig fat tissue of the obese group revealed the direct role of MALAT1 expression in an obesity-related context^57^. The precise mechanisms explaining positive interactions between increased CSI and MALAT1 expression on VAI and BAI cannot be definitively determined based on the current study. Nonetheless, it is important to consider various possibilities derived from experimental investigations. Based on an animal model study, increased expression of MALAT1 in the livers of genetically leptin-deficient obese (ob/ob) mice exposed to palmitate was demonstrated to result in hepatic lipid accumulation^49^. Certainly, under conditions characterized by fatty acid overload, such as with palmitate, there is an observed elevation in intracellular levels of triglycerides and cholesterol in primary mouse hepatocytes^58^. It has also been suggested that high-fat diets in obese mice trigger a set of dysregulated lncRNAs in adipocytes which increases fat mass through decreasing plasma leptin^59^. Another possible mechanism for lipid profile incremental changes depends on the direct link between MALAT1 expression with *peroxisome proliferator-activated receptor* γ (PPARγ) and PPARγ coactivator 1-α (PGC1α)^60^. PPARγ is a nuclear receptor, which is activated by PGC1α, and displays tendencies for lipid synthesis and fat accumulation^61–63^. Furthermore, the occurrence of metabolic disorders seems to be attributed to the elevated expression of PPARγ induced by a high-fat diet.^64^. On the other hand, gene silencing of MALAT1 in mice significantly attenuated the palmitate-induced lipid accumulation of both intracellular levels of triglycerides and cholesterol^49^. Another possible explanation accounting for the observed interaction might be attributed to the absence of MALAT1, which seems to reduce 3-hydroxy-3-methylglutaryl coenzyme A (HMG-CoA) reductase, the rate-limiting enzyme of cholesterol synthesis^49^. Notably, reducing MALAT1 expression in the liver of ob/ob mice did not exhibit any impact on body weight^49^. Moreover, a recent study revealed that suppressing MALAT1 did not influence the accumulation of adipose tissue in mice induced by either age or dietary factors^65^. Regarding TUG1, our study did not identify any links between TUG1 expression and CSI affecting VAI and BAI. The possible candidate explaining our findings could be microRNA-204 (miR-204), a key tumor-suppressor microRNA that engages in the process of obesity^66^. It has been documented that miR-204 was abnormally up-regulated in obese mice and its overexpression is more likely to inhibit the expression of TUG1^67^.

Consistent with our results, no notable correlation was observed between TUG1 and obesity-related parameters in VAT when accounting for age and HOMA-IR^50^. However, obese mice on a high-fat diet exhibited multiple essential functions of TUG1 in reducing weight and lipid accumulation. This effect was accomplished by stimulating the expression of leptin and adiponectin^67^.

Until recently, the extent to which long non-coding RNAs (lncRNAs) are implicated in the genetic mechanisms governing metabolic homeostasis and disorders, particularly in the context of addressing obesity and influencing the aforementioned outcomes, remained largely unexplored. It is also noteworthy that the majority of the studies are conducted on animal models and it can be proposed as one possible reason for these contradictory results. As such, our study along with others could uncover the possible role of MALAT1 and TUG1 associated with CSI on VAI and BAI among overweight/obese women. However, several limitations deserve to be mentioned. Firstly, the use of cross-sectional study design precludes the extrapolation of the results. Furthermore, environmental factors such as physical activity and nutrient status affecting lncRNAs have not been explored. The use of FFQ for recording the individual's food, which is subjected to recall bias, can also be regarded as a limitation. Given the significant roles of adipokines, particularly leptin and adiponectin, in the pathogenesis of obesity and related disorders, it is crucial to assess their connections with the two studied lncRNAs. As this study exclusively involved women, the generalizability of our findings to all genders is limited. Consequently, it is strongly recommended to conduct further studies with larger sample sizes encompassing both men and women to comprehensively address this issue.

## Conclusion

In summary, this study represents the inaugural attempt to assess the interplay of the long non-coding RNAs (lncRNAs), MALAT1 and TUG1, in relation to CSI and its impact on VAI and BAI. The findings suggest a positive correlation between MALAT1 expression and CSI, potentially influencing VAI and BAI. Conversely, no robust associations were detected between TUG1 and the aforementioned outcomes. Nonetheless, further investigations in human studies are imperative to validate and expand upon these concepts in this field.

### Supplementary Information

Supplementary Table S1.

## Data Availability

The data supporting the findings of this study are not publicly available due to restrictions imposed by the license under which they were used for the current study. However, interested parties may request access to the data from the authors, with permission from the corresponding author, and subject to reasonable conditions.
