# Supplementary material for: The interaction between MALAT1 and TUG1 with dietary fatty acid quality indices on visceral adiposity index and body adiposity index
Source: Sci Rep. 2024 Jan 2;14:12. doi: 10.1038/s41598-023-50162-9 (PMC10762150; doi:10.1038/s41598-023-50162-9)
Supplement: Supplementary file 1 — Supplementary Table S1. [file 41598_2023_50162_MOESM1_ESM.docx]

| **Table S1: Forward and reverse primers used for real-time PCR.** | | |
| --- | --- | --- |
| Primer | Forward sequence | Reverse sequence |
| MALAT1 | 5'-GACGAGTTGTGCTGCTATCTT-3' | 5'-GATTCTGTGTTATGCCTGGTTAG-3' |
| TUG1 | 5'-TAGCAGTTCCCCAATCCTTG-3' | 5'-CACAAATTCCCATCATTCCC-3' |
| 18s rRNA | 5′- GGAGAGGGAGCCTGAGAAAC-3′ | 5′- CAATTACAGGGCCTCGAAAG-3′ |
| MALAT1, metastasis-associated lung adenocarcinoma transcript 1; TUG1, taurine upregulated gene 1. | | |
